# Prevalence and risk factors of trematode infection in swamp buffaloes reared under different agro-climatic conditions in Java Island of Indonesia

**DOI:** 10.14202/vetworld.2020.687-694

**Published:** 2020-04-15

**Authors:** Nanis Nurhidayah, Fadjar Satrija, Elok Budi Retnani, Dewi Apri Astuti, Sri Murtini

**Affiliations:** 1 Parasitology and Medical Entomology Study Program, Graduate School of IPB University, Bogor, Indonesia; 2 Department of Animal Infectious Diseases and Veterinary Public Health, Faculty of Veterinary Medicine, IPB University, Bogor, Indonesia; 3 Department of Animal Nutrition and Feed Technology, Faculty of Animal Science, IPB University, Bogor, Indonesia

**Keywords:** epidemiology, Indonesia, swamp buffalo, trematodes

## Abstract

**Background and Aims:**

This study was conducted to estimate the prevalence and intensity and to identify the associated risk factors and impact of trematode infection in swamp buffaloes reared under different agro-climatic conditions in Java, Indonesia.

**Materials and Methods:**

A total of 580 fecal samples were collected from swamp buffaloes in five different agro-climatic areas in Banten and Central Java Provinces, Indonesia. The fecal samples were examined using the Danish Bilharziasis Laboratory Technique to determine the prevalence and intensity of trematode infection. The risk factors for infection were determined from an in-depth interview of owners/keepers, and the results were analyzed using Chi-square tests and multiple logistic regression. The infection was also correlated with swamp buffalo production parameters (body weight and body condition score [BCS]).

**Results:**

From all fecal samples, the overall prevalence of trematode infection was 64.83%, which comprised *Fasciola* spp. (16.03%; mean eggs per gram [EPG]±SD: 1.02±0.43) and Paramphistomatidae (62.93%; mean EPG±SD: 1.01±0.66). The main risk factor for trematode infection was feeding animals with rice straw (odds ratio [OR]: 40.124); the risk of trematode infection was 40.142 times higher in buffaloes that consumed rice straw. Other risk factors included the frequency of anthelmintic treatment (OR: 4.666), age (OR: 0.449), and drinking water source (OR: 0.358). Trematode infection did not significantly affect the body weight or BCS of swamp buffaloes.

**Conclusion:**

Although the prevalence of trematode infection was high in swamp buffaloes, the intensity of infection was low, and the infection did not affect the animals’ physical parameters.

## Introduction

Swamp buffalo (*Bubalus bubalis*) play an important role in the agricultural economy in most developing Asian countries by providing meat, farm animal labor, and a source of organic fertilizer [1]. In Indonesia, the animals are also part of social culture, for example, in family savings and as sacrificial animals in traditional and/or religious ceremonies [2]. In 2018, the swamp buffalo population in Indonesia was 1,932,927 heads; these animals were geographically distributed in Sumatera, Java, Kalimantan, and Sulawesi, as well as in West and East Nusa Tenggara [3]. Swamp buffaloes in seven regions of Indonesia (Aceh, Riau, Madiun, Blitar, Lombok, South Kalimantan, and Tana Toraja) share similarities in genetics and their management systems. The majority of these livestock are reared under traditional husbandry systems characterized by a lack of knowledge and technology [4]. The previous studies have reported trematode infection in buffaloes worldwide [5-7]. Although infected animals may not exhibit any clinical signs, infection reduces livestock performance, for example, by slowing growth rate, lowering body weight, and reducing milk production [8,9]. In the acute form of trematode infection, extensive organ damage and even sudden death can occur due to the migration of high numbers of immature flukes [10].

Studies on trematode infection in Indonesian buffalo have been very limited, although a high prevalence was reported in South Kalimantan, Aceh, and Central Sulawesi [11-13]. The disease situation potentially differs across the country since Indonesia is divided into 14 agro-climatic regions, according to the number of annual wet and dry months accumulated over 10 years of observation [14].

Here, we conducted a study of swamp buffalo in several districts and provinces in Java Island of Indonesia with the following aims: (1) To estimate the prevalence of trematode infection, (2) to identify risk factors for trematode infection, and (3) to determine the impact of trematode infection on the production performance of livestock. This study is important because we are accumulating the fundamental data required for future control of trematode infection in Indonesian swamp buffaloes within and outside of the study location.

## Materials and Methods

### Ethical approval

The procedures applied throughout this study have been approved by the Animal Care and Use Committee of IPB University No.65-2017 IPB and 133-2018 IPB.

### Study area

**Figure-1:**
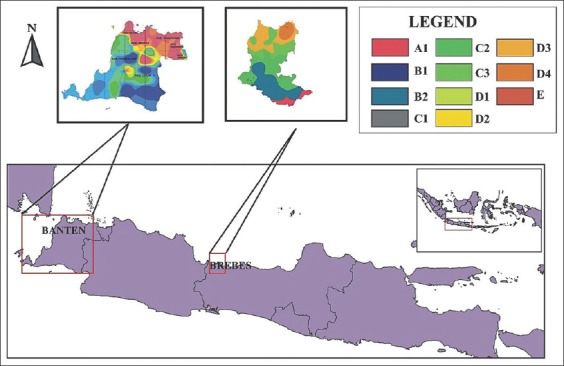
Agro-climatic map of study locations (the map was re-drawn by Nanis Nurhidayah using Oldeman classification Maps from Pondok Betung Climatology Station and Semarang Climatology Station).

A cross-sectional study was carried out from March 2017 to May 2018 in 12 sub-districts of Indonesia, including eight in the Districts of Serang and Lebak, Banten Province, and four in Brebes District, Central Java Province. The study areas were categorized into five different agro-climatic types based on recent data of the Oldeman climate map from the nearest climate stations (Table-1 and Figure-1).

**Table-1:** Agro-climatic types of the study sites based on the Oldeman classification map.

Agro-climatic region type	Number of consecutive		Location

	Wet months	Dry months	
B2	7-9	2-4	Panggarangan, Lebak District, Banten Bantarkawung, Brebes District, Central Java
C1	5-6	<2	Cipanas, Lebak District, Banten Cinangka, Serang District, Banten
C2	5-6	2-4	Cinangka, Serang District, Banten Mancak, Serang District, Banten Salem, Brebes District, Central Java Tonjong, Brebes District, Central Java
C3	5-6	5-6	Anyer, Serang District, Banten Cikulur, Lebak District, Banten Cileles, Lebak District, Banten Cinangka, Serang District, Banten
D4	3-4	>6	Brebes, Brebes District, Central Java

### Sample and data collection procedures

Fecal samples were randomly collected from 580 swamp buffaloes, including groups of pre-weaned calves (0-8months), calves (>8-18months), young animals (>18-32months), and adults (>32months). Aminimum fecal sample of 15g was collected from each animal. The samples were placed in labeled plastic containers, stored in a cooler box, and transported to the Laboratory of Helminthology, Department of Animal Infectious Diseases and Veterinary Public Health, Faculty of Veterinary Medicine, IPB University.

Subsequently, the production parameters of the fecal-sampled animals, including their body condition score (BCS) and girth, were evaluated [15]. BCS was estimated by measuring eight skeletal checkpoints. Measurements of girth can be used to estimate body weight by ANIMETER^®^; girth data were transformed into kilograms [16].

An in-depth interview conducted through a structured questionnaire was targeted at 317 owners/keepers to assess the potential risk factors of trematode infection. The interview included questions on animal and owner status, management systems, and animal health management.

### Coprological technique

Fecal samples were examined using the Danish Bilharziasis Laboratory (DBL) technique [17] with slight modifications. Specifically, 5-g samples were weighed, added to 10ml of water, homogenized, and filtered using 400-µm, 100-µm, and 45-µm sieves. The fecal material that remained on the 45-µm sieve was collected into a sedimentation tube, which was refilled with water. The sample was then centrifuged 2000rpm for 10minutes, and water was added to make a volume of 2.25ml. From this mixture, a 150-µl aliquot of each sample was added to a counting slide. The number of trematode eggs per gram (EPG) in fecal samples was obtained from three counting slides. The animal was considered to be positively infected by trematodes, if trematode eggs were observed during the microscopic examination. The prevalence of infection was calculated for total samples, each parasite, and each category (i.e.,age group, sub-district, sex, and type of agro-climate). The infection intensity was determined based on EPG and the geometric mean.

### Statistical analysis

The prevalence and intensity of infection were represented by descriptive statistics, with 95% exact confidence intervals (CIs). Risk factors were analyzed using Chi-square tests and multiple logistic regressions. AKendall’s tau-b correlation was run to determine the relationships between infection intensity and production parameters. All statistical procedures were processed in SPSS v.16 (IBM Corp., NY, USA) for Windows.

## Results

### Prevalence and infection intensity

**Figure-2:**
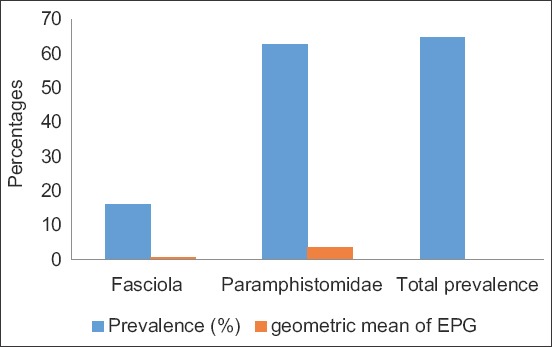
Prevalence and intensity of trematode infection in Indonesian swamp buffaloes.

The results of the coprological examination showed that 376 of 580, i.e.,64.83% (CI: 60.94-68.71%), swamp buffaloes had excreted trematode eggs. Two trematode egg types were identified microscopically: *Fasciola* spp. in 16.03% (CI: 13.05-19.02%) of samples and Paramphistomatidae in 62.93% (CI: 59.00-66.86%). The infection intensity of *Fasciola* spp. and Paramphistomatidae was 0.74 EPG (CI: 0.19-1.29 EPG) and 3.60 EPG (CI: 2.07-5.13 EPG), respectively (Figure-2).

The prevalence and infection intensity of *Fasciola* spp. according to several variables is shown in Table-2. The prevalence and infection intensity of *Fasciola* spp. was higher in female buffaloes (17.26%, CI: 13.86-20.66%; 0.79 EPG, CI: 0.71-1.41 EPG) than in males (10.48%, CI: 4.62-16.33%; 0.46 EPG, CI: 0.26-0.65 EPG). According to age group, the highest trematode prevalence and infection intensity occurred in adult buffaloes, followed by calves, pre-weaned calves, and young animals, respectively. Of 110 swamp buffaloes had a BCS of 2.5, 32 were infected by *Fasciola* spp. with an infection intensity of 0.56 EPG (CI: 0-1.19 EPG).

**Table-2:** Prevalence and intensity of *Fasciola* spp. infection in Indonesian swamp buffaloes.

Variables	Sample size (%)	Number of positive samples	Prevalence (95% exact CI)	Geometric mean EPG (95% exact CI)
Sub-districts				
Anyer	12 (2.07)	2	16.67 (0-37.75)	0.43(0-0.92)
Cinangka	105 (18.10)	28	26.67 (18.21-35.13)	0.54 (0.09-1.00)
Mancak	22 (3.79)	2	9.09 (0-21.10)	0.25 (0)
Padarincang	11 (1.9)	1	9.09 (0-26.08)	0.50 (0)
Cipanas	47 (8.1)	10	21.28 (9.58-32.98)	0.45 (0-0.93)
Cikulur	37 (6.38)	3	8.11 (0-16.90)	0.66 (0-1.89)
Cileles	19 (3.28)	5	26.32 (6.52-46.12)	0.59(0.17-1.01)
Panggarangan	87 (15)	16	18.39 (10.25-26.53)	0.65 (0-1.69)
Salem	51 (8.79)	5	9.80 (1.64-17.97)	1.32 (0.34-2.30)
Bantarkawung	90 (15.52)	9	10.00 (3.80-16.20)	1.36 (0.70-2.02)
Tonjong	69 (11.9)	9	13.04 (5.10-20.99)	2.32 (0-7.01)
Brebes	30 (5.17)	3	10.00 (0-20.74)	1 (0)
Sex				
Male	105 (18.1)	11	10.48 (4.62-16.33)	0.46 (0.26-0.65)
Female	475 (81.9)	82	17.26 (13.86-20.66)	0.79 (0.71-1.41)
Age				
0-8 months	56 (9.66)	7	12.50 (3.84-21.16)	0.43 (0.31-0.56)
>8-18 months	56 (9.66)	8	14.29 (5.12-23.45)	0.45 (0-2.09)
>18-32 months	81 (13.97)	6	7.41 (1.70-13.11)	0.57 (0.21-0.93)
>32 months	387 (66.72)	72	18.60 (14.73-22.48)	0.80 (0.46-1.15)
BCS				
2	5 (0.86)	0	0	0
2.5	110 (18.97)	32	29.09 (20.60-37.58)	0.56 (0-1.19)
3	295 (50.86)	44	14.92 (10.85-18.98)	0.71 (0-1.72)
3.5	148 (25.52)	15	10.14 (5.27-15.00)	1.38 (0.34-2.42)
4	22 (3.79)	2	9.09 (0-21.10)	0.78 (0.43-2.57)
Agro-climatic area				
B2	237 (40.86)	32	13.50 (9.15-17.85)	0.78 (0.22-1.35)
C1	35 (6.03)	7	20.00 (6.75-33.25)	0.41 (0-1.10)
C2	185 (31.9)	36	19.46 (13.75-25.16)	0.89 (0-2.19)
C3	93 (16.03)	15	16.13 (8.65-23.60)	0.53 (0.10-0.95)
D4	30 (5.17)	3	10.00 (0-20.74)	1 (0)

EPG=Eggs per gram, CI=Confidence interval

We made interesting observations related to trematode infection in various sub-districts and agro-climatic areas. Although the highest prevalence of *Fasciola* spp. was observed in the Cinangka sub-district, the swamp buffaloes in Tonjong excreted more eggs than those in any other area. Similarly, the swamp buffaloes in agro-climatic area C1 had a slightly higher prevalence of infection (20%, CI: 6.75-33.25%) than buffaloes in other areas, but the highest infection intensity of *Fasciola* spp. was found in the agro-climatic area D4.

As shown in Table-3, the prevalence and infection intensity of Paramphistomatidae was higher compared with those of *Fasciola* spp. across all variables; however, there was a similarity in the infection pattern. Paramphistomatidae was more prevalent in females, adults, and buffaloes with a BCS of 2.5. In the district of Anyer, all swamp buffaloes tested was infected by Paramphistomatidae (100%); whereas buffaloes in Tonjong excreted a higher rate of eggs in their feces (18.51 EPG, CI: 12.34-24.67 EPG). When analyzing infection according to the agro-climatology of study areas, Paramphistomatidae prevalence was high in all areas (>60 %), but the highest intensity was recorded in the agro-climatic area C2.

**Table-3:** Prevalence and intensity of paramphistomatida in Indonesian swamp buffaloes.

Variables	Sample size (%)	Number of positive samples	Prevalence (95% exact CI)	Geometric mean EPG (95% exact CI)
Sub-districts				
Anyer	12 (2.07)	12	100 (100-100)	2.48 (0-5.20)
Cinangka	105 (18.10)	68	64.76 (55.26-73.90)	1.88 (0-4.19)
Mancak	22 (3.79)	7	31.82 (12.36-51.28)	0.67 (0.31-1.03)
Padarincang	11 (1.9)	2	18.18 (0-40.97)	1 (0)
Cipanas	47 (8.1)	35	74.47 (62.00-86.93)	2.87 (1.35-4.38)
Cikulur	37 (6.38)	27	72.97 (58.66-87.28)	1.41 (0-4.09)
Cileles	19 (3.28)	17	89.47 (75.67-100)	2.60 (0.08-5.12)
Panggarangan	87 (15)	76	87.36 (80.37-94.34)	3.99 (1.27-6.70)
Salem	51 (8.79)	15	29.41 (16.91-41.92)	3.14 (0-7.87)
Bantarkawung	90 (15.52)	30	33.33 (23.59-43.07)	3.15 (0-6.71)
Tonjong	69 (11.9)	54	78.26 (68.53-87.99)	18.51 (12.34-24.67)
Brebes	30 (5.17)	22	73.33 (57.51-89.16)	6.19 (0-12.73)
Sex				
Male	105 (18.1)	49	46.67 (37.12-56.21)	2.41 (0-7.04)
Female	475 (81.9)	316	66.53 (62.28-70.77)	3.83 (2.21-5.45)
Age				
0-8 months	56 (9.66)	22	39.29 (26.49-52.08)	0.96 (0-2.18)
>8-18 months	56 (9.66)	28	50.00 (26.90-63.10)	2.05 (0-7.57)
>18-32 months	81 (13.97)	38	46.91 (36.05-57.78)	3.50 (0-7.93)
>32 months	387 (66.72)	277	71.58 (67.08-76.07)	4.20 (2.74-5.94)
BCS				
2	5 (0.86)	4	80.00 (44.94-100)	0.79 (0-1.88)
2.5	110 (18.97)	88	80.00 (72.52-87.48)	3.19 (1.38-5.00)
3	295 (50.86)	180	61.02 (55.45-66.58)	2.83 (0.99-4.67)
3.5	148 (25.52)	78	52.70 (44.66-60.75)	8.11 (3.28-12.95)
4	22 (3.79)	15	68.18 (48.72-87.64)	2.93 (0-9.50)
Agro-climatic areas				
B2	237 (40.86)	146	61.60 (55.41-67.80)	3.25 (1.54-4.95)
C1	35 (6.03)	24	68.57 (53.19-83.95)	1.77 (0-3.69)
C2	185 (31.9)	112	60.54 (53.50-67.58)	6.01 (2.22-6.01)
C3	93 (16.03)	61	65.69 (55.94-75.25)	1.96 (0-4.47)
D4	30 (5.17)	22	73.33(57.51-89.16)	6.19 (0-12.73)

BCS=Body condition score, EPG=Eggs per gram, CI=Confidence interval

### Risk factors for trematode infection

Results of Chi-square analysis indicated that sex, age, availability of wallow, straw feeding, drinking water source, and frequency of anthelmintic treatment were all significantly related to infection (p<0.05). In contrast, variation in agro-climate did not significantly impact trematode infection (Table-4). The odds ratio (OR) of animals that were fed with rice straw was 40.124, which meant that these animals were 40.124times more likely to be infected with trematodes. In addition, trematode infection increased 4.666-fold in animals that received no anthelmintic treatment (OR: 4.666). An age-dependent pattern was also observed for OR values (Table-5): Adult swamp buffaloes were most likely to be infected (OR: 0.449), followed by young animals (OR: 0.350), calves (OR: 0.084), and pre-weaned calves (used as a reference).

**Table-4:** Chi-square analysis of trematode infection in Indonesian swamp buffaloes.

Variables	Number of non-infected buffalo	Number of infected buffalo	p-value
Sex			0.000^a^
Male	32	29	
Female	71	185	
Age			0.001^a^
Pre-weaned calves	17	12	
Calves	14	16	
Young	18	28	
Adult	54	158	
Wallow			0.032^a^
No	73	125	
Yes, available	30	89	
Rice-straw feeding			0.011^a^
	2	21	
Yes	101	193	
Drinking water-source			0.003^a^
Tap water	1	6	
Pond	99	176	
Mix	3	32	
Frequency of anthelmintic treatment			0.014^a^
Monthly	48	71	
Sick only	29	96	
None	26	47	

a Statistical significance (p<0.05)

**Table-5:** Multivariate analysis of risk factors for trematode infection in Indonesian swamp buffaloes.

Variables	Odds ratio	95% CI	p-value
Rice-straw feeding			
No		Reference	
Yes	40.124	2.438-660.413	0.010
Frequency of anthelmintic treatment			0.000
Monthly		Reference	
Sick only	0.773	0.256-2.329	0.647
None	4.666	1.686-12.912	0.003
Age group			0.000
Pre-weaning calves		Reference	
Calves	0.084	0.028-0.257	0.000
Young animals	0.350	0.124-0.991	0.048
Adults	0.449	0.200-1.008	0.052
Drinking water source			0.004
Tap water		Reference	
Pond	0.358	0.011-12.174	0.568
Mix source	0.028	0.003-0.246	0.001
Constant	7.528		0.086

CI=Confidence interval

### Trematode infection impact correlated with production parameters

The results of Kendall’s tau-b correlation analysis for variables are shown in Table-6. Alow-positive correlation was observed between the two production parameters, BCS and body weight (p<0.05). However, there was no significant correlation between the intensity of trematode infection and the production performance parameters. The EPG of *Fasciola* spp. was not significantly correlated with either BCS or body weight; in contrast, both body weight and BCS were positively correlated with Paramphistomatidae EPG (Kendall’s tau-b correlation coefficient: 0.237 and 0.254, respectively, (p<0.001).

**Table-6:** Correlation coefficients for trematode infection correlated with production performance parameters in Indonesian swamp buffaloes.

	BCS	BW	EPG *Fasciola* spp.	EPG Paramphistomatidae
BCS	1.000	0.125^a^	−0.022	0.237^b^
BW		1.000	0.069	0.254^b^
EPG *Fasciola* spp.			1.000	0.196^b^
EPG Paramphistomatidae				1.000

a Statistically significant (p<0.05),

b Statistically significant (p<0.01), BCS=Body condition score, BW=Bodyweight, EPG=Eggs per gram

## Discussion

The high level of trematode infection we observed in Indonesian swamp buffaloes is in agreement with reports from other areas of the country [12,13]. The prevalence of fasciolosis in Indonesian swamp buffaloes tends to be higher than in the riverine types from India and Pakistan [18,19]. The wet tropical environment consists of the perfect temperature and humidity for infection, which is likely the primary determining factor for this variation in prevalence among countries.

In this study, we used the DBL technique to collect data; the method is simple, it does not require special reagents or equipment, nor does it require toxins, and it enables clear observation of trematode eggs. The most practical limitations of this technique were the number of feces samples required and the time required for preparation and analysis. Nevertheless, the DBL technique is essential for both the detection and quantification of trematode eggs in buffaloes, dogs, cattle, and pigs in the Philippines and Indonesia [20,21]. Using this technique, we found two different egg types belonging to *Fasciola* spp. and Paramphistomatidae, a finding that is in accord with studies of buffalo and beef cattle in Central Sulawesi and Bojonegoro, East Java [21,22].

Swamp buffaloes play an important role as the main definitive host of *Fasciola gigantica* in Indonesia. Mukhlis [23] suggested that *F. gigantica* is the only endemic *Fasciola* species that infects ruminants in Indonesia; his hypothesis is based on the limited availability of the specific intermediate host snail in Indonesia, namely, *Lymnaea rubiginosa*. According to molecular characterization and phylogenic analysis, *F. gigantica* was the only species found in West Java, which is closed to *Fasciola* found in other Asian countries. As yet, no hybrid *Fasciola* has been detected in Indonesia unlike in most other Asia countries [24].

In this study, the identification of parasites was based on the morphology of their eggs on microscopic examination. The distinctive characteristic of *F. gigantica* eggs compared with eggs from other members of *Fasciola* (e.g.,*Fasciola hepatica*) is their size: *F. gigantica* eggs are larger (90-100 µm×170-190µm) than *F. hepatica* eggs (63-79 µm×126-133µm) [23]. Liver fluke (i.e.,*Fasciola*) eggs are yellowish-brown, whereas the eggs of rumen flukes are much lighter in color. The size of Paramphistomatidae eggs is around 75-100 µm×115-175µm and buffaloes can be infected by the following species from the Paramphistomatidae family: *Paramphistomum cervi* and *Cotylophoron cotylophorum* [25]. Unfortunately, the microscopic examination technique used in this study was not precise enough to allow identification at the species level.

Of the trematode infections observed in this study, those from Paramphistomatidae were most prevalent and intense. This is likely related to the numerous species of Paramphistomatidae that infect Asian cattle and buffalo, as well as the variety and prevalence of its intermediate host, i.e.,freshwater snail species from the families Planorbidae and Lymnaeidae [26]. The dominance of Paramphistomatidae over *Fasciola* spp. may also be explained by the location in which their cercariae encyst. The cercariae of *F. hepatica* encyst on the water surface and are, therefore, lost during heavy rain and/or flooding, whereas *Paramphistomum daubneyi* encyst at the bottom of water bodies near to the soil and develop to be the infective stage, i.e.metacercariae. Furthermore, soil provides the perfect level of moisture to maintain the development of *P. daubneyi* metacercariae [27].

Although the prevalence of both parasites was high in the present study, the infection intensity was relatively low. The observed EPG of Paramphistomatidae could be associated with the actual number of adult parasites in the abdominal region; however, thousands of adult rumen flukes may not induce clinical signs in their host [28]. In contrast, the low EPG of *Fasciola* spp. is likely caused by the trapping of eggs before they enter the intestinal lumen or by an over dispersion factor [29]. Evidence of an overdispersion also found in the previous case of fasciolosis in the cattle, so that highly infected animals excreted a very low number of eggs in their fecal sample [30]. Unfortunately, the low-infected animals could play an important role as an infection source for animals at the herd level since all of the respondents of this study said that they placed their buffaloes in communal cages and shared grazing areas.

Chi-square analysis revealed that there was no correlation between agro-climatic variation and parasitic infection in buffalo. This finding was similar to that of an epidemiological study of parasitic gastrointestinal infection on sheep, which took place over a year in various climatic areas in West Java, Indonesia [31]. Alack of correlation might have been the result of similarities between the five agro-climatic areas studied here, which are all categorized within the “wet climate region” group. The main risk factor for trematode infection in the present study was feeding animals with rice straw, which increased the risk of infection up to 40.124times. This is unsurprising since most of the metacercariae of *F. gigantica* are reported to attach to the lower part of the rice stalk close to the soil (0-10cm) and to inundate these plants [32]. Cattle and buffalo potentially became infected as rice fields were plowed or during grazing around the harvesting period. At the same time, buffaloes drink from and wallow in water sources; buffaloes that used ponds as a water source were 0.358times more likely to become infected than those that used other water sources.

The age of swamp buffaloes was also a decisive factor in their infection status. Adults were at most risk of infection, and the likelihood of infection decreased with age. This result supports previous findings in cattle and buffalo from Pakistan [33]. The long prepatent period of the parasites and the high exposure of adult buffaloes to the pre-parasitic stages when the animals are used to plow paddy fields are thought to be the main causes of the increased risk in this age group [34]. Since the farmers kept adults for breeding purposes, more than 80% of the swamp buffalo population consisted of adults. This infection situation was aggravated further by a lack of anthelmintic therapy, which was shown to increase trematode infection 4.666-fold.

Correlation analysis revealed that the production performance of swamp buffaloes in the study areas was not affected by trematode infection. In contrast to our findings, *Fasciola* spp. induced negative effects on the body condition of riverine-type buffaloes in Egypt [35]. On the other hand, an experimental study of Indonesian buffalo infected by *F. gigantica* reported similar results to those in our study [36]. Agroup of *Fasciola*-infected buffalo calves exhibited similar daily weight gains to those of control animals, whereas *Fasciola*-infected Bali and Ongole calves added less daily weight than animals in a control group. An improved immune response, including the number of eosinophilia, Th2-related cytokines, and/or effective digestion and fermentation in the front abdomen, is known to play an important role in the initial response to these parasites [37,38]. On the other hand, the traditional semi-intensive system allows buffaloes to select their preferred feed, which potentially has anthelmintic properties (e.g.,condensed tannins, alkaloids, glycosides, allicin, and santonin) [39]. Therefore, further analysis of the identity and content of forage consumed by buffaloes during the grazing period is required in the study location.

## Conclusion

The parasitization of swamp buffaloes by *Fasciola* spp. and Paramphistomatidae is highly prevalent in different agro-climatic areas of Java Island, Indonesia, although the infection intensity in these areas is relatively low. Major risk factors for infection are feeding animals with rice straw, the frequency of anthelmintic treatment, animal age, and drinking water source. The infection did not produce a significant negative impact on the production performance of swamp buffaloes. Nevertheless, control measures are needed to prevent the future spread of the disease in herds.

## Authors’ Contributions

NN, FS, EBR, DAA, and SM designed the study. NN collected and examined the fecal samples. FS helped to perform statistical analysis, data interpretation, and the drafting process. EBR and SM cosupervised the laboratory examination. DAA contributed to the analysis of the impact of trematode infection against production performance parameters as well as revising and editing the manuscript. All authors read and approved the final manuscript.
